# Chylothorax Following Thoracolumbar Corpectomy: Successful Conservative Management With Octreotide: A Case Report

**DOI:** 10.7759/cureus.91909

**Published:** 2025-09-09

**Authors:** Shogo Mitamura, Shinya Takahashi, Toru Tominaga, Masahiro Yoshida

**Affiliations:** 1 Orthopaedics, Seirei Mikatahara General Hospital, Hamamatsu, JPN

**Keywords:** chyle in the pleural cavity, chylothorax, lateral lumbar corpectomy, postoperative complication, retropleural approach, thoracolumbar spine surgery

## Abstract

An 84-year-old woman developed delayed-onset neurological deficits due to a T12 osteoporotic vertebral fracture. She underwent anterior corpectomy and reconstruction of the T12 vertebral body via a retropleural approach. A standard surgical drain was placed and removed on postoperative day (POD) 7. Thereafter, progressive left-sided pulmonary infiltrates were observed. On POD 14, a chest drain was reinserted due to increasing pleural effusion. The drainage fluid appeared turbid yellow, and pleural fluid analysis showed a triglyceride level of 117 mg/dL, consistent with a diagnosis of chylothorax. Despite the initiation of a fat-restricted diet, chyle output persisted. On POD 30, octreotide was initiated at 100 µg subcutaneously three times daily for 6 days. The drainage ceased by the third day of treatment.

This case highlights the possibility of chylothorax even in procedures that do not involve the mediastinum. Injury to an aberrant thoracic duct at the thoracolumbar junction may explain this rare postoperative complication. Octreotide therapy was effective in resolving the chyle leak after conservative dietary management failed.

Chylothorax can occur after retropleural approaches to the thoracolumbar spine. Awareness of this rare complication and prompt initiation of medical treatment, including somatostatin analogues such as octreotide, may allow successful nonoperative management.

## Introduction

Chylothorax, defined as the accumulation of lymphatic fluid in the pleural cavity, is a rare but potentially serious complication of spine surgery with an estimated incidence of <0.5% overall [1]. While most commonly associated with thoracic or mediastinal procedures, it has occasionally been reported following anterior spinal surgeries, particularly those involving retroperitoneal or retropleural approaches [2,3]. The presumed mechanism involves inadvertent injury to lymphatic structures such as the thoracic duct or its tributaries, which may be more susceptible to disruption at the thoracolumbar junction due to anatomical variability [4].

While chyle leaks in spinal surgery have occasionally been reported in the retroperitoneal space [2,3], their extension into the pleural cavity, resulting in chylothorax, remains a rare occurrence. The clinical presentation may be subtle and delayed, and without early recognition and intervention, patients may develop nutritional, immunological, and respiratory complications [5].

We report a unique case of isolated left-sided chylothorax following a T12 vertebral body replacement via a retropleural approach. This case highlights the potential for thoracic duct injury even in procedures that avoid the mediastinum and emphasizes the importance of early recognition and conservative management strategies, including the use of somatostatin analogues [6].

Objective: We report this case to emphasize the importance of early recognition and to highlight octreotide as a valuable adjunct to conservative management.

## Case presentation

An 84-year-old woman presented with progressive lower extremity weakness and bladder dysfunction secondary to a T12 osteoporotic vertebral fracture. Neurological examination revealed motor weakness graded as 3/2 in bilateral hip and knee flexion/extension and 3/3 in ankle dorsiflexion and plantarflexion. She had a complete loss of walking ability and urinary incontinence. Her Japanese Orthopaedic Association (JOA) score for thoracic myelopathy was 2, and her JOABPEQ subscores were: pain-related disorders 100, lumbar function 8.3, walking ability 0, and mental health 50.5.

The patient’s comorbidities included hypertension, osteoporosis, and chronic heart failure. Her baseline nutritional status was impaired, with BUN 36 mg/dL and creatinine 0.6 mg/dL, suggesting relative catabolism.

**Figure 1:**
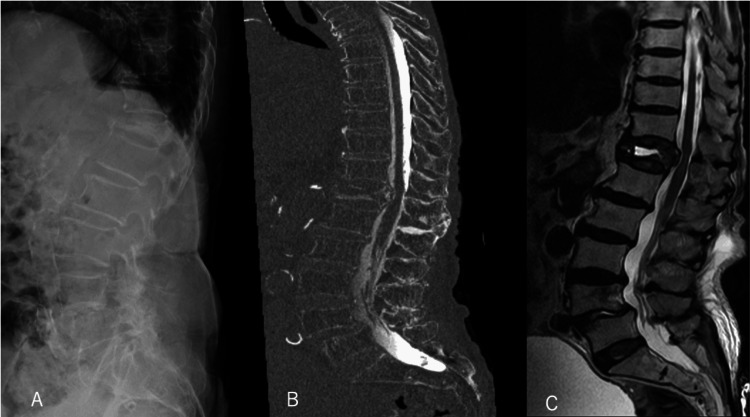
Preoperative Imaging A: Lateral radiograph showing a T12 compression fracture. B: CT myelography showing severe spinal canal stenosis. C: T2-weighted MRI showing intravertebral high signal intensity.

Preoperative imaging demonstrated a T12 compression fracture with canal stenosis and spinal cord signal change (Figure 1).

The patient underwent thoracolumbar vertebral body replacement via a left-sided retropleural approach, followed by posterior instrumentation. Intraoperatively, there was partial injury to the parietal pleura but no injury to the visceral pleura or visible chyle leakage. A standard retropleural drain was placed and removed on postoperative day (POD) 7.

**Figure 2:**
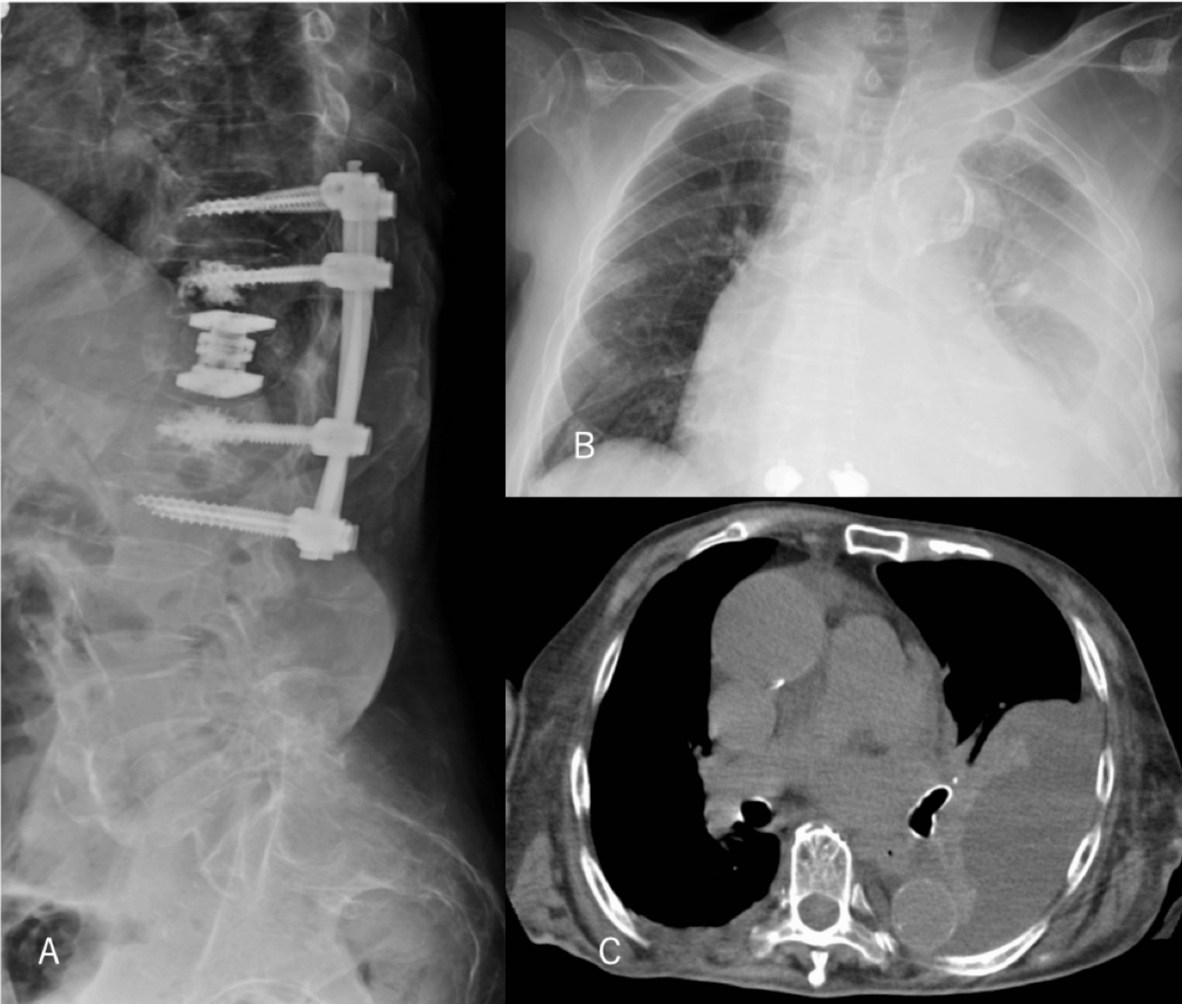
Postoperative Imaging and Development of Pleural Effusion A: Postoperative lateral radiograph demonstrating vertebral body replacement at T12 and posterior instrumentation. B: Chest radiograph on postoperative day 14 showing progressive left-sided pleural effusion. C: Chest CT confirming a large left pleural effusion. Complete resolution of the pleural effusion was confirmed on follow-up chest radiograph after octreotide therapy (not shown).

Postoperative radiographs confirmed implant placement, but subsequent imaging demonstrated progressive left pleural effusion (Figure 2).

**Figure 3:**
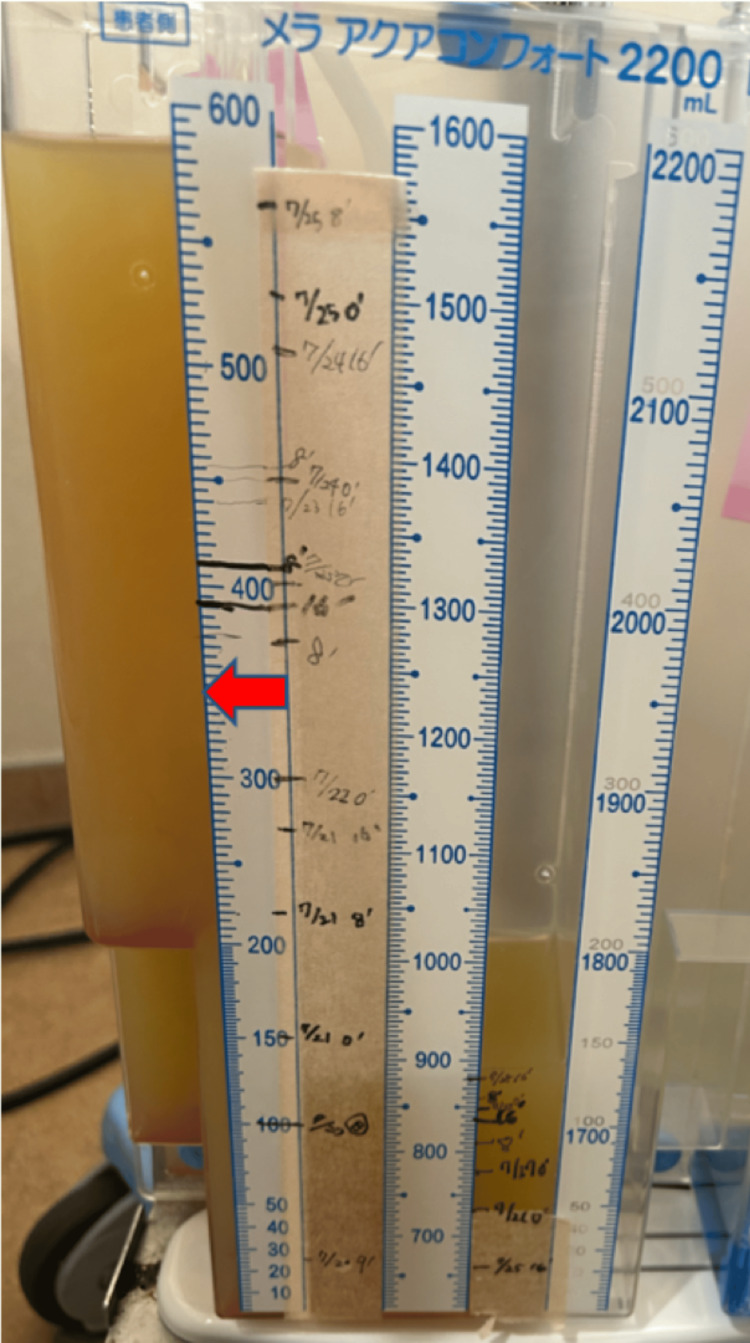
Appearance of Pleural Drainage in Chylothorax Photograph of the pleural drainage bag taken on postoperative day 14. The turbid yellow fluid (indicated by the red arrow) is consistent with chylothorax.

On POD 14, a thoracic drain was reinserted due to increased left-sided effusion. The pleural fluid appeared turbid yellow (Figure 3).

Biochemical analysis revealed a triglyceride level of 117 mg/dL, a total protein concentration of 2.3 g/dL, and lactate dehydrogenase (LDH) of 159 U/L. The glucose level was 150 mg/dL, with a concurrent serum glucose of 129 mg/dL. Cytological analysis showed a lymphocyte predominance of 64.3%. Bacterial cultures were negative. These findings were consistent with an exudative effusion and met the criteria for chylothorax [5,7].

Despite initiating a fat-restricted diet, chyle leakage persisted, with daily outputs of approximately 100-250 mL. Octreotide initiation was delayed until POD 30 because the drainage remained in the low-to-moderate range, and conservative management was initially pursued. On POD 30, octreotide therapy was initiated at a dose of 100 µg subcutaneously, three times daily. By POD 33, the drainage ceased entirely and remained at 0 mL/day thereafter. The chest drain was removed on POD 41. 

**Figure 4:**
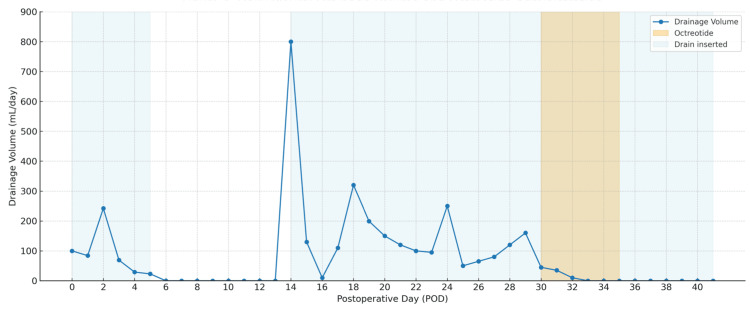
Daily Pleural Drainage Volume and Octreotide Administration Graph showing daily pleural drainage volumes over the postoperative course. Blue shading indicates periods when a thoracic drain was in place. Orange shading marks the duration of octreotide therapy, which was initiated on postoperative day 30 and led to the rapid resolution of chyle output. The image is created by the author.

Figure 4 illustrates the daily drainage volume and the timing of octreotide administration.

A chest X-ray taken after six days of octreotide therapy showed complete resolution of the effusion. The patient remained clinically stable and was planned for transfer to a rehabilitation facility. Follow-up after transfer to rehabilitation showed no recurrence of chylothorax at six months, recovery of serum albumin levels, and gradual improvement in daily activity independence.

## Discussion

Chylothorax is a rare but recognized postoperative complication following anterior spinal surgery, typically resulting from unintentional damage to the thoracic duct or one of its tributaries [2,3]. It most commonly occurs after transthoracic or retroperitoneal approaches, particularly in the upper thoracic spine, where the thoracic duct is more prominent and vulnerable [4]. However, chylothorax following retropleural exposure at the thoracolumbar junction is exceedingly rare, with very few cases reported in the literature. Only a handful of thoracolumbar chylothorax cases have been reported in the literature (fewer than 10 cases to date), most following anterior or anterolateral approaches to the thoracolumbar junction [1-4]. Some reports indicate improvement with conservative treatment, while others suggest that surgical treatment was required [1-4]. Surgical treatments described include thoracic duct ligation, chemical pleurodesis, and interventional radiology-guided thoracic duct embolization, which are generally reserved for cases refractory to conservative or pharmacologic management [6,7]. In contrast, cases managed with octreotide have shown successful resolution without the need for reoperation, as was the case in our patient.

The thoracic duct originates at the cisterna chyli, located near the L2 vertebral level, and ascends through the posterior mediastinum. It typically runs to the right of the aorta, then crosses to the left at around the T5-T6 level before terminating at the left venous angle. However, numerous anatomical variations in the course, branching, and duplication of the thoracic duct have been described. In particular, the lower thoracic duct and its accessory branches may course more laterally or have aberrant paths that place them at risk during anterior dissection, especially in minimally invasive or retropleural approaches that limit direct visualization of lymphatic structures.

Chylothorax is generally diagnosed when the pleural fluid triglyceride level exceeds 110 mg/dL, with additional supportive findings such as lymphocyte-predominant cytology and sterile cultures [5,7-9]. In this case, the triglyceride level of 117 mg/dL, lymphocyte fraction of 64.3%, total protein concentration of 2.3 g/dL, and negative cultures fulfilled these accepted diagnostic criteria. Although the fluid was not milky in appearance, the biochemical profile indicated chylothorax.

Initial conservative management included drainage, a fat-restricted diet, and peripheral parenteral nutrition. However, the output remained persistently high, exceeding 100-200 mL/day for over two weeks. On postoperative day 30, we initiated therapy with octreotide at 100 µg subcutaneously three times per day. Octreotide, a somatostatin analogue, reduces lymphatic flow by inhibiting splanchnic circulation and decreasing intestinal absorption of fats. Its use in the treatment of chyle leaks, particularly in thoracic duct injuries and postoperative chylothorax, has been increasingly reported [6,10,11], although strong evidence remains limited to case series and observational studies. Reported dosing regimens vary widely, ranging from 50 µg every 8 hours to continuous infusions of 500 µg/day for 5-14 days, with reported success rates of 60-80% [12-14].

Following the initiation of octreotide, drainage volume decreased rapidly and ceased entirely within three days. No recurrence was observed, and the thoracic drain was removed on postoperative day 41. This clinical course is clearly illustrated in Figure 4, demonstrating a temporal association between pharmacologic intervention and resolution of the leak.

This case highlights several important clinical points. First, chylothorax can develop even in spinal procedures that do not directly involve the mediastinum, owing to the anatomical variability of lymphatic pathways at the thoracolumbar junction. Second, the absence of a milky appearance in pleural fluid does not rule out chylothorax, particularly in patients who are receiving modified diets or are under catabolic stress. Third, octreotide may serve as a safe and effective adjunct in the conservative management of persistent chyle leaks, potentially avoiding the need for surgical re-exploration.

Given the aging population and increasing use of anterior approaches for vertebral reconstruction, awareness of this rare but significant complication is essential. Early recognition and timely initiation of nonoperative therapies may improve outcomes and reduce hospital stay. Limitations include the single-patient nature, limited follow-up, absence of direct thoracic duct imaging, and lack of pleural cholesterol measurement. Further studies are needed to establish standardized protocols for the diagnosis and treatment of postoperative chylothorax in spinal surgery.

## Conclusions

Chylothorax is a rare but possible complication even in anterior spinal surgeries that avoid direct mediastinal manipulation, such as retropleural approaches at the thoracolumbar junction. Variations in thoracic duct anatomy may place lymphatic structures at risk during dissection, even when the procedure remains extramediastinal. This case highlights the importance of considering chylothorax in the differential diagnosis of postoperative pleural effusions, even when the fluid is not overtly milky. This case also demonstrates that escalation to octreotide can achieve resolution after dietary management fails.

Conservative treatment with a fat-restricted diet may be insufficient in persistent cases, and adjunctive therapy with somatostatin analogues such as octreotide can be effective in achieving resolution without the need for reoperation. Early recognition and prompt therapeutic intervention may improve patient outcomes and shorten hospital stays. Clinical implication: Spine surgeons should recognize that chylothorax may occur even after retropleural approaches and consider octreotide as an effective non-surgical option.
